# Migration of breast cancer cell lines in response to pulmonary laminin 332

**DOI:** 10.1002/cam4.957

**Published:** 2016-11-22

**Authors:** Philip M. Carpenter, Priyanka Sivadas, Spencer S. Hua, Cally Xiao, Alyssa B. Gutierrez, Tuan Ngo, Paul D. Gershon

**Affiliations:** ^1^ Department of PathologyKeck School of Medicine, the University of Southern CaliforniaLos AngelesCalifornia; ^2^ Department of PathologyUniversity of CaliforniaIrvineCalifornia; ^3^ Department of Pharmacology and Experimental TherapeuticsUniversity Hospital of CologneCologneGermany; ^4^ St. George’s UniversitySt. GeorgeGrenada; ^5^ Department of Molecular Biology and BiochemistryUniversity of CaliforniaIrvineCalifornia

**Keywords:** Laminin 332, pulmonary epithelium, tumor cell migration, tumor microenvironment

## Abstract

Because tumor cell motility is a requirement for metastasis, we hypothesized that lung tissue harbors substances that induce tumor cell migration. MCF‐7 breast carcinoma cells exposed to small airway epithelial cells and conditioned medium exhibited dose‐dependent tumor cell migration. Among the extracellular matrix proteins in the conditioned medium identified by mass spectrometry, laminin 332 (LM332) had the greatest contribution to the migration of MCF‐7 cells. Immunoblotting and immunohistochemistry for LM332‐specific chains identified LM332 in the lung and in pulmonary epithelial cells. Antibodies to either LM332 or its integrin receptor inhibited MCF‐7 motility, and knockdown of LM332 chains also reduced its migration‐inducing activity. Taken together, these findings implicate LM332 as a component of lung tissue that can induce motility in breast carcinoma cells that have been transported to lung during metastasis. Earlier studies on LM332 in tumor progression have examined LM332 expression in tumor cells. This investigation, in comparison, provides evidence that the tumor promoting potential of LM332 may originate in the lung microenvironment rather than in tumor cells alone. Furthermore, this study provides evidence that the motility‐inducing properties of the microenvironment can reside in epithelial cells. The findings raise the possibility that LM332 plays a role in the pulmonary metastases of breast carcinoma and may provide a target for antimetastasis therapy.

## Introduction

Metastasis is almost always the cause of death for cancer patients, yet little is known about its mechanisms, and the inhibition of metastasis remains a major challenge of oncology. Metastasis is a multi‐step process in which transformed cells must separate from neighboring healthy cells, invade the stroma, enter and exit the vasculature, and invade into and grow in a different tissue type from their tissue of origin 1. This process may be simplified into two major steps, namely physical translocation and colonization 1. Animal studies have indicated that colonization is relatively inefficient 1, 2, and therefore might be highly dependent upon the target microenvironment for the metastasis to become established. The requirement for enhanced migration of tumor cells for metastasis has been demonstrated in several model systems 3, 4. In many cases, the tumor microenvironment itself is the source of migratory signals. These motility factors include cytokines 5, 6, chemokines 5, and extracellular matrix (ECM) proteins 7, 8, 9. Therefore, the study of tumor cell migration and the lung microenvironment and molecules within it is relevant to the study of metastasis.

The lung is often the first site of metastasis for breast carcinoma, especially among the clinically aggressive basal subtype of breast cancer 10, 11. The mechanisms responsible for the propensity of certain cancer types to metastasize to particular organs remain a mystery. Paget hypothesized over 100 years ago that microenvironmental signals in the target organ were responsible for the establishment of a metastasis, with cancer cell “seeds” requiring specific “soil” in which to grow 11, 12. A few mechanisms have been proposed to explain how this might happen. Recently, the concept of the premetastatic niche has been proposed 13, whereby a cancer anywhere in the body can induce bone marrow precursor cells to migrate to the lung, where they secrete cytokines and other factors that attract tumor cell micrometastases. Alternatively, normal pulmonary cells such as pneumocytes, bronchial epithelium, endothelium, and stromal cells may secrete substances that might serve as motility or growth factors. Similarly, ECM proteins found in the pulmonary interstitium or basement membrane may function to attract tumor cells 14 and physical properties of the ECM such as stiffness may also affect tumor progression 14. Many studies of tumor progression have focused on the roles of inflammatory 13, endothelial 15, and/or stromal cells 14 in the tumor microenvironment. Epithelial cell contributions may occur as well, particularly after the tumor cells have undergone transendothelial migration.

Laminin 332 (LM332), also known as laminin 5, is among ECM components implicated in tumor migration, invasion, and metastasis 16, 17, 18, 19. Laminins are large extracellular glycoproteins that are expressed by basal epithelium and are important components of basement membranes 20. Laminins are heterotrimers containing *α*,* β*, and *γ* chains forming a cross‐shaped structure 20. LM332 consists of *α*3, *β*3, and *γ*2 laminin chains 18. The major functions of LM332 include mediating the binding of epithelial cells to the basement membrane through the formation of hemidesmosomes 21, and inducing the migration of epithelial cells during wound repair 17, 22. LM332 is known to stimulate the migration of various cells including carcinoma cells 16, 17. LM332 expression has been shown to correlate with tumor invasiveness and poor patient prognosis 18. LM332 also is noted to accumulate at the interface of the tumor with the surrounding stroma or in the cytoplasm of cancer cells 23.

This study was undertaken to assess whether LM332 produced by pulmonary small airway epithelial cells (SAEC) influence breast carcinoma migration, using an in vitro model. This study extends the work of prior studies on LM332 by examining its potential role as a microenvironmental inducer of migration of tumor cells in the lung.

## Materials and Methods

### Tissue culture of breast cell lines

Tissue culture medium components and other chemicals were obtained from Sigma Chemicals (St. Louis MO) except where otherwise indicated. MDA‐MB‐231 cells were obtained as a gift from Dr. Noo Li Jeon and MCF‐7 cells were obtained as a gift from Dr. Dan Mercola. The human mammary cell lines BT‐20, SKBR3, and 184A1 24 were obtained from the American Type Culture Collection (Rockville, MD). The verification of cell lines by identification of their short tandem repeat sequences was performed by the Molecular Pathology Research Core at the University of Southern California. MCF‐7 cell medium consisted of RPMI 1640 medium supplemented with 4 mmol/L glutamine, 0.2 U/mL bovine insulin, 10 U/mL penicillin, 10 *μ*g/mL streptomycin, 5% heat‐inactivated FBS (Gemini Scientific, Calabasas CA), and 10 nmol/L estradiol. BT‐20, SKBR3, and MDA‐MB‐231 were maintained in RPMI 1640 with 4 mmol/L glutamine, 10 U/mL penicillin, 10 *μ*g/mL streptomycin, and 10% FBS. 184A1 cells were routinely grown in mammary epithelial growth medium (MEGM) (Lonza, Walkersville, MD). MCF‐7 cells for imaging by fluorescence microscopy were transfected with green fluorescent protein (GFP) DNA using the pEGFP‐C1 vector (BD Bioscience, San Jose CA) and selected using G418 at 1 mg/mL followed by flow cytometry with cell sorting to isolate brightly green fluorescent tumor cells.

### Purification of pulmonary small airway epithelial cells

Normal, anonymous human lung tissue removed at surgery was obtained from the University of California (UCI) Pathology Tissue Biorepository, according to a UCI Human Subjects Committee protocol. Tissue was minced and rinsed in PBS and then incubated with trypsin for 30 min. The resulting digest was suspended in RPMI 1640 in a 15 mL centrifuge tube and cells were allowed to settle for 5 min. The supernatant was removed and the settled lung cells were plated in RPMI containing 10% FBS, and allowed to adhere to the tissue culture plastic. After 2 days at 37°C, the medium was changed to small airway growth medium (SAGM) (Lonza). Confluent cells were typically obtained within 2 weeks. An additional source of cells for these experiments was purified small airway epithelial cells purchased from Lonza. These cells were seeded at 2500 cells per cm^2^ in SAGM and propagated according to the supplier's instructions. For both sources of SAEC, the cells showed epithelial morphology in culture and were positive for cytokeratin by immunohistochemistry (AE1/AE3, Leica Biosystems, Richmond IL, pre diluted).

### Growth of small airway epithelial cells at a liquid–air interface

Purified SAEC were grown on membranes in Bronchial –Air Liquid Interfaces Differentiation Medium (B‐ALI), (Lonza) for 3 days, then transferred in B‐ALI at a density of 240,000 cells per cm^2^ on to transparent polyester membranes with 0.4 micrometer pores (Millipore, Bedford MD). Four to 17 h later, after the cells had attached to the membranes, the B‐ALI covering the cells was removed, and fresh B‐ALI was placed in the wells beneath the membranes to provide a membrane–air interface mimicking the normal lung microenvironment, as has been previously described 25. Cells on membranes were used for experiments after 7 or 8 days of growth at the liquid–air interface.

### Collection of conditioned medium from SAEC

SAEC at 50% to 100% confluency remained viable for up to 1 week in SAGM. The SAGM was collected, and cell debris was removed with a 0.2‐*μ*m pore syringe filter. Enrichment for ECM proteins and 30‐fold concentration of the CM was performed by ultrafiltration with Amicon Ultra concentrators with a 100 kDa cutoff (Millipore).

### MCF‐7 scattering assay

Scattering of MCF‐7 cells is due to the separation and migration of individual cells from clusters of cells, as we have previously shown using time‐lapse video microscopy 26. The effect of lung‐conditioned medium (CM) on MCF‐7 cell scattering was measured by adding the CM to wells of 96‐ or 48‐well tissue culture plate containing 2 × 10^4^ MCF‐7 cells per mL. As negative controls, the same volume of SAGM was added in place of CM. Experiments were terminated after overnight incubation, by rinsing the cells briefly with PBS and staining with 2% crystal violet in 40% ethanol. The proportion of motile cells was determined by a semiquantitative method 26, 27. Briefly, wells of crystal violet‐stained MCF‐7 cells were divided into half, and the total number of cells in a single cluster of 20–80 cells in predetermined coordinates was counted for each half well. The number of cells that had separated from the main group and exhibited lamellipodia or pseudopodia, were counted in each of these clusters to determine the number of motile cells per colony. The average proportion of motile cells per condition and the standard error were calculated from these counts. Unless otherwise specified, for all scattering assays, RPMI or PBS in a volume equal to the volume of CM or purified protein was the negative control, and 184A1 CM, which has motility‐inducing properties 16, was the positive control. All assays were performed in at least triplicate wells and repeated at least once.

### Coculture of isolated SAEC and MCF‐7 cells

Pulmonary airway cells in MCF‐7 medium were diluted to 1000, 500, 250, 100, or 50 SAEC per well of a 96‐well tissue culture plate in duplicate wells. SAEC were given 24 h at 37^°^C to attach. Following incubation, MCF‐7 medium in each well was removed and replaced with 2 × 10^3^ MCF‐7 cells in 0.1 mL of medium. After 24 h at 37^°^C, cells were stained with 2% crystal violet and scattering was measured as described above.

For visualization of MCF‐7 interactions with pulmonary epithelium, SAEC were labeled with 1 μg/μL SNARF^®^‐1 carboxylic acid, acetate succinimidyl ester (Invitrogen, Thermo‐Fisher Scientific, Waltham, MA) for one hour, followed by transfer of the cells in SAGM onto glass chamber slides at approximately 50% confluency. After overnight incubation to allow the cells to adhere to the glass, GFP‐labeled MCF‐7 cells were added in 50% SAGM and MCF‐7 medium. The use of the vital dye and GFP allowed imaging of living cells. After another overnight incubation, cells were visualized and photographed with a Zeiss LSM510 fluorescent microscope, with AIM software (Carl Zeiss MicroImaging, Inc., Jena, Germany). Excitation and transmission settings for GFP were 488 and 505 nm, respectively, and settings for SNARF^®^‐1 carboxylic acid, acetate succinimidyl ester were 488 and 560 nm, respectively.

### Motility assay of CM components bound to tissue culture plastic

Filter‐sterilized, concentrated lung CM in SAGM was added to duplicate wells of a 96‐well tissue culture plate in the following amounts: 100, 50, 25, 10, 5, 1, and 0 *μ*L per well, and all wells were then brought to an equal volume using PBS. After overnight incubation at 37°C to allow binding of CM components to the bottom of the wells, the wells were rinsed and MCF‐7 cells were added as above. After an additional overnight incubation, motility of the MCF‐7 was measured. SAGM concentrated 30‐fold by ultrafiltration using an Amicon Ultra concentrator with a 100 kDa cutoff (Millipore, Bedford, MA) served as a negative control.

### Boyden chamber assay

Either 30× concentrated lung CM, or an equal volume of SAGM as a negative control was allowed to incubate overnight on the lower surface of a Millicel polycarbonate membrane (Millipore) with 12‐*μ*m diameter pores. In triplicate wells of a 24‐well tissue culture plate, 10^5^ BT‐20, SKBR3, or MDA‐MB‐231 cells were placed on one side of the membrane. The total volume of each well was 1 mL of RPMI with 1% FBS with 0.5 mL on each side of the membrane. After 6 h, nonmigrating cells were removed with cotton‐tipped swabs, so that they did not obscure the view of the migrating cells. Motile cells that migrated through the pores to the other side of the membrane were fixed and stained with 2% crystal violet in 40% ethanol. The average number of cells per high power field was counted in triplicate for each of the membranes, resulting in a total of nine fields for each condition. The standard error and unpaired Student's *t*‐test were calculated using Microsoft Excel.

### Mass Spectrometry

In order to determine the identity of molecules from CM bound to tissue culture plastic, two different extraction methods were used to remove the molecules from the plastic. First, after overnight incubation of wells of a 24‐well tissue culture plate were with 30× concentrated lung epithelial CM, bound protein was extracted using 1% sodium lauryl sulfate (SDS) and 0.2% deoxycholate buffer. Scattering assays of duplicate wells that were not extracted confirmed that the migration‐inducing substances were bound to the plastic. MCF‐7 cells added to the wells after extraction no longer exhibited scattering, confirming the removal of motility‐inducing substances. SDS‐deoxycholate‐extracted samples were supplemented with SDS to 4% and then subjected to filter‐assisted sample preparation (FASP) 28. Peptides trypsinized from the FASP membrane (1:50 trypsin:substrate) were isolated using C18 STAGE‐tips 29. Eluted, vacuum‐dried peptides were redissolved in 10 *μ*L of 0.1% formic acid in water for nanoLC‐MS/MS, using an LTQ mass spectrometer (Thermo‐Fisher) with Waters 600 HPLC running with split flow. The five most intense precursor peptides per spectrum were subjected to fragmentation and, using Mascot 2.3, the resulting peaklist against a SwissProt database with human taxonomy was searched. In addition, a decoy search was performed using a database of common contaminants.

### Dose–response analysis of purified ECM proteins

To determine the dose response of MCF‐7 cells to the ECM proteins identified by mass spectrometry in CM, the scattering assay was performed using recombinant transforming growth factor β‐induced protein ig‐h3 (βig‐h3) at concentrations ranging from 0.27 to 5.5 *μ*mol/L, thrombospondin 1 (TSP1) at 0.15 to 77.5 *μ*mol/L, fibronectin from 0.04 to 20 *μ*mol/L, recombinant tenascin C at 1.5 to 300 *μ*mol/L, recombinant laminin 511 (LN511) at 0.025 to 12.8 *μ*mol/L, and purified LM332 at 0.5 to 100 *μ*mol/L. Equal volumes of PBS served as a negative control. The recombinant βig‐h3 and tenascin C were purchased from R&D Systems (Minneapolis, MN), recombinant LN511 was purchased from BioLamina (Stockholm, Sweden), fibronectin (Superfibronectin) was purchased from Sigma, and the LM332 was purified from 184A1 CM as previously described 16.

### Immunoblot

Crude lung CM harvested after 2 days of incubation was concentrated 60‐fold, boiled in reducing Laemmli buffer, and applied to an 8% SDS‐PAGE gel. After electrophoresis, the proteins were transferred to a nitrocellulose membrane and incubated with a mouse polyclonal antibody against *α*3 laminin chain (Abnova, Taipei, Taiwan) diluted 1:500, in blotto buffer, and monoclonal antibodies against *β*3 laminin chain (clone 17/kalinin B1 Transduction Laboratories, Lexington KY) diluted 1:1000 at 0.25 *μ*g/mL and γ2 laminin chain (clone D4B5 Millipore) diluted 1:800–2 *μ*g/mL. Where applicable, *β* actin Abcam8226 (Abcam, Cambridge, MA) was diluted 1:1000–23 *μ*g/mL. After incubation, washing and application of a biotinylated goat anti‐mouse secondary antibody (Thermo‐Fisher Scientific) diluted 1000 times (0.8 mg/*μ*L), the signals were developed with the Pico Super Signal West chemoluminescent detection system (Thermo‐Fisher Scientific) according to the manufacturer's instructions and bands were visualized with the C‐Digit scanner (Licor, Lincoln, NE).

### Immunohistochemistry

Two of the antibodies used for immunoblotting were suitable for immunohistochemistry. Sections of formalin‐fixed human lung tissue or cultured SAEC on membranes at a liquid–air interface were paraffin‐embedded and stained for the LM332 *β*3 and *γ*2 chains as described previously 30. Each 4‐*μ*m thick section on capillary gap slides were deparaffinized with Histoclear (National Diagnostics, Atlanta GA). Sections were then rehydrated through decreasing concentrations of isopropyl alcohol. Immunohistochemical reactions were performed using a Ventana BenchMark Ultra automated immunostainer (Ventana Medical Systems, Inc., Tucson, AZ). The automated steps included pH 6 citrate‐buffered antigen retrieval, blockage of endogenous peroxidase, and reactions with the primary antibody, secondary antibody, and linkage to peroxidase. The chromogen was diaminobenzidine for all reactions.

Immunoperoxidase reactions were performed using the anti‐kalinin B1 monoclonal antibody (Transduction Laboratories), which reacts with the *β*3 laminin chain, diluted 1:250 (1 *μ*g/mL) in PBS and the *γ*2 chain diluted 1:20 (50 *μ*g/mL). Negative controls were performed in the same fashion, except that the primary antibody was substituted with mouse immunoglobulin. A section of breast tissue served as the positive control. Microscopy and photography were performed using an Olympus (Center Valley, PA) BX 46 microscope with a DP71 camera and DP Controller and DP Manager photography software.

### Neutralizing antibody assay

Incubation of 20 *μ*L of 30× concentrated lung CM in wells of a 96‐well plate overnight at 4^°^C allowed binding of the motility‐inducing substances of the CM to the bottom of each well. A total quantity of 4.5 *μ*g of LM332 was allowed to bind to separate wells overnight and served as a control. Media and LM332 were removed, the wells rinsed with PBS, and 5 *μ*g anti‐LM332 blocking antibody P3H9 (Millipore) were added to duplicate wells. After 4 h of incubation at 4^°^C, the antibody was removed, the wells were rinsed, and 2000 MCF‐7 cells in growth media were added. After overnight incubation at 37^°^C, scattering assays were performed. For integrin blocking studies, wells with bound motility factor from lung CM or bound LM332 as prepared above were rinsed with PBS, and 20 *μ*g/mL of blocking antibodies against *α*3 integrin clone P1B5 (Millipore), *β*1 integrin clone 6SC (Millipore), or purified mouse immunoglobulin and 2000 MCF‐7 cells were added to wells. After overnight incubation at 37°C, scattering assays were performed on two randomly chosen colonies of cells from each well.

Each individual experimental condition (mouse Ig, anti‐ LM332, anti‐*α*3 integrin, and anti‐*β*1 integrin) was carried out for either lung 30× concentrated CM or LM332 20 *μ*g/mL. Six replicates were performed for each individual experimental variable condition. Individual experimental variable condition with means ± standard error of the mean was analyzed by one‐way ANOVA with Holm–Sidak correction for multiple comparisons. Statistical analyses were performed in Stata 12 software (StataCorp. 2011, College Station, TX).

### LM332 chain knockdown

In wells of a 48‐well plate, SAEC cells were transfected with equal amounts of siRNA designed to target *LAMA3, LAMB3,* and *LAMC2* genes. The sequences of the RNAs, (SA Biosciences, now Qiagen Germantown, MD) were as follows: CCAGCUCACCUGUGUCUACAA, GACAGGAGAUUCCAGCUUCAA, and GCUGGAGUUUGACACGAAUAU, respectively. A random negative control sequence of ACACUAAGUACGUCGUAUUAC was used at the same concentration as the total concentration for the three laminin RNAs. For each well, 1.5 *μ*L of HiPerfect transfection reagent (Qiagen) was preincubated for 10 min with 10 fmole of total siRNA, in 50 *μ*L of small airway basal medium (without bovine serum albumin or other additions). Each condition was performed in triplicate wells. Subsequently, 50,000 second passage SAEC cells were added to each well with the siRNA complex and incubated for 3 h at 37^°^C. An additional 200 *μ*L of SAGM was then added to each well. The final concentrations of reagents were therefore 33.3 nmol/L siRNA and 1.5 *μ*L transfection reagent per 300 *μ*L incubation. After 48 h at 37^°^C in 5% CO_2_, 100 *μ*L of the conditioned medium from each well was transferred to a new well of a second 48‐well plate for a scattering assay. A total of 10,000 MCF‐7 cells were added to each well and migration was measured. The transfected SAEC cells remaining in the wells were lysed with 50 *μ*L of SDS gel loading buffer, and SDS‐PAGE using equal amounts of lysate was performed for each condition. The protein bands were then transferred to nitrocellulose membrane, and immunoblotting was performed as described above using the same antibodies against the *α*3, *β*3, and *γ*2 laminin chains, with *β* actin as a loading control. In some experiments, only *LAMA3* siRNA was added using the same conditions and immunoblot and motility assays were performed as described above. All knockdown experiments were repeated at least once.

## Results

### Motility induced by cultured lung epithelium

**Figure 1:**
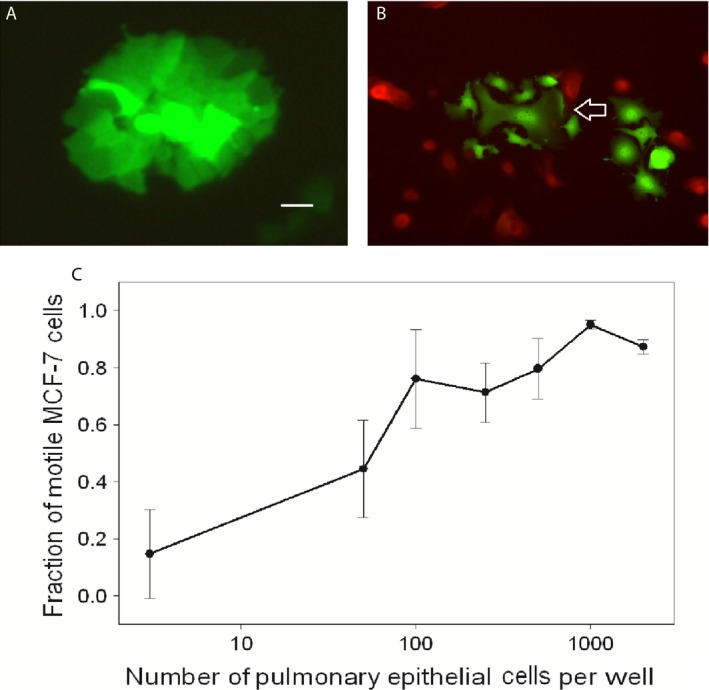
MCF‐7 cells transfected with GFP grown in standard culture conditions (A), and with SAEC labeled red with SNARF ^®^‐1 carboxylic acid, acetate succinimidyl ester (B). MCF‐7 cells separate from the clusters and display pseudopodia and lamellipodia (arrow). Original magnification 400×, scale bar = 50 *μ*m. MCF‐7 scattering at different densities of SAEC (C), revealing dose‐dependent scattering with increasing numbers of SAEC cells. Error bars show standard errors of the mean. SAEC, small airway epithelial cells; GFP, green fluorescent protein.

We aimed to test the hypothesis that epithelial cells such as pneumocytes and bronchiolar cells from lung tissue produce factors that have the capacity to induce breast cancer cell migration. Insofar as lung tissue is a combination of cell types including pneumocytes, bronchial epithelium, stromal cells, and endothelium, we focused on the role of epithelial cells isolated from lung tissue and grown in culture. To determine whether coculture of SAEC and MCF‐7 could induce motility in the breast carcinoma cells, increasing numbers of SAEC labeled red with SNARF^®^‐1 carboxylic acid, acetate succinimidyl ester were cocultured with GFP‐labeled MCF‐7 and scattering assays were performed. The use of these labels allowed visualization of living MCF‐7 and SAEC cocultures undergoing the migratory phenotype by fluorescence microscopy. MCF‐7 cells cultured in the absence of SAEC were not motile (Fig. 1A), however, the addition of SAEC induced scattering of MCF‐7 (Fig. 1B), characterized by MCF‐7 cells separating from the clusters and displaying pseudopodia and lamellipodia. Moreover, the motility response was dose‐dependent (Fig. 1C), with increasing MCF‐7 scattering with increasing numbers of SAEC cells. Thus, the pulmonary epithelial cells were a source of motility‐inducing properties from the lung.

### Motility induced by SAEC‐conditioned medium

**Figure 2:**
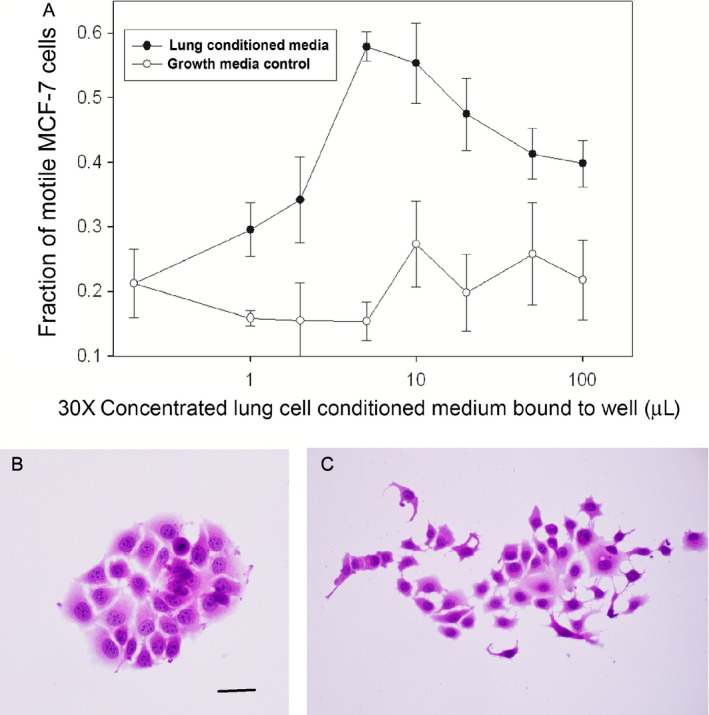
Dose‐dependent scattering of MCF‐7 in the presence of increasing of conditioned medium (CM) of small airway epithelial cells (SAEC) (A). Nonmotile MCF‐7 cells in the absence of SAEC CM (B) and displaying a scattering response in the presence of 20 *μ*L of SAEC CM (C). Hematoxylin and eosin stain, scale bar = 50 *μ*m.

**Figure 3:**
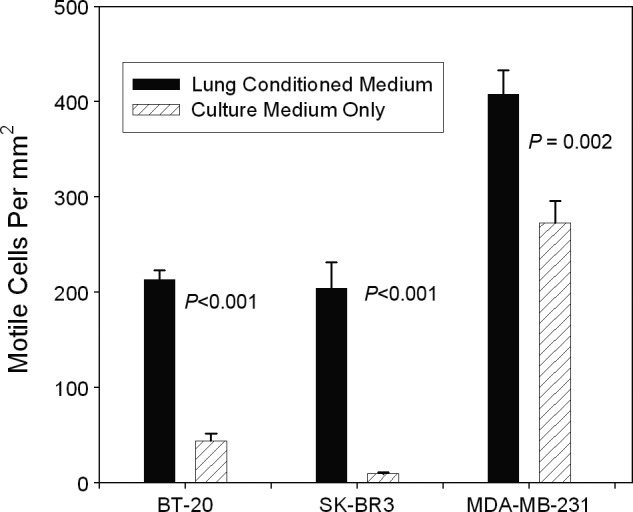
Boyden chamber assays showing significant induction of motility by small airway epithelial cells (SAEC) CM in three breast carcinoma cell lines. Error bars show standard errors of the mean.

To determine whether the motility induced by SAEC was due to a secreted material, CM was collected from cultures of SAEC and molecules heavier than 100 kDa were concentrated 30‐fold. As a control, SAGM underwent the same concentration process. Serial dilutions of the CM were allowed to incubate overnight in wells of tissue culture plastic plates, followed by removal of the media and unbound molecules. MCF‐7 cells showed a similar dose‐dependent migratory phenotype in response to the SAEC motility factor deposited on the plastic (Fig. 2) as was seen with SAEC cocultures. Scattering is greatest at approximately 8–10 *μ*L of conditioned medium per well compared to equal amounts of growth medium concentrated to the same degree as the CM. Thus, SAEC secreted a motility‐inducing substance that remained active while attached to cell culture plastic and after subsequent addition of MCF‐7 cells. Using a Boyden chamber assay, the mammary carcinoma cells lines MDA‐MB‐231, BT‐20, and SKBR3 exhibited a migratory phenotype in the presence of SAEC CM (Fig. 3), evidenced that CM from lung induces motility in these other breast carcinoma cell lines.

### Identification of the deposited motility factor

To determine protein constituents of the deposited material from CM, an overnight incubation of CM on tissue culture plastic was performed. Coated wells were rinsed and deposited protein was extracted using SDS/deoxycholate buffer. To confirm that the extraction was complete, MCF‐7 cells were added to the well after the extraction, and decreased motility was observed, in contrast to wells incubated with CM but not extracted. The extracted material was digested with trypsin and subjected to mass spectrometry. The experiment was repeated, except that extraction was performed with trypsinization directly in the well, followed by a second mass spectrometry analysis of tryptic peptides. One hundred and eight and 84 protein families were identified as a false discovery rate of 5% in the respective experiments. Tables 1 and 2 show results after exclusion of likely contaminants and hits with Mascot scores of below 30. Many of the proteins identified appeared to be nuclear or cytoplasmic in origin, such as cytoskeletal proteins and/or housekeeping enzymes, most likely arising from lysed or dead cells. Among the proteins with the highest Mascot scores were ECM proteins (Table 3), namely fibronectin, TSP1, *β*ig‐h3, tenascin C, and laminin *α*3, *β*3, *γ*2 *α*5, *β*1, and *γ*1 chains. The laminin chains were the components of LM332 (laminin 5) and LM511 (laminin 10). These proteins have all been implicated in tumor cell motility 16, 17, 31, 32, 33, 34, 35. The confident identification of pulmonary surfactant‐associated protein B provided confirmation that the cultured cells were of pulmonary epithelial origin.

**Table 1:** Summary of proteins of the SDS‐deoxycholate (first) extraction detected by mass spectrometry

Mascot score	Protein family	Peptide matches
308	**Thrombospondin‐1**	11
278	Desmoplakin	10
247	**Tenascin**	10
194	**Transforming growth factor‐** ***β*** **‐induced protein ig‐h3**	8
181	Hemoglobin subunit beta, human	3
144	Pyruvate kinase, Isozymes M1/M2	2
132	**Laminin ** ***α*** **3 chain**	4
131	Sulfhydryl oxidase 1	5
129	Moesin	4
100	Ezrin/radixin	4
128	Actin	4
104	Lactate dehydrogenase A chain	3
102	14‐3‐3 protein zeta/delta	1
100	Desmocollin	2
97	**Laminin ** ***β*** **3 chain**	2
95	Lysozyme	1
79	Fructose‐bisphosphate aldolase A	1
78	Hornerin	2
73	Follistatin	1
68	Glyceraldehyde‐3‐phosphate dehydrogenase	2
68	Phosphoglycerate kinase	2
68	Actin, aortic smooth muscle	1
65	Elongation factor 1‐alpha 1	2
63	Cathepsin D	2
61	Heat shock protein HSP 90‐alpha	1
60	Renin receptor	1
57	Annexin A1	1
55	S100‐P	1
55	Heat shock cognate 71	2
52	Annexin A2	1
49	Dynein heavy chain 12	2
49	ATP‐binding cassette subfamily G member 2	1
48	Apolipoprotein D	1
48	Galectin 7	1
47	Mitofusin‐2	1
46	Glutathione S‐transferase P	1
46	Pulmonary surfactant‐associated protein B	1
45	S100‐A9	2
44	V‐type proton ATPase subunit S1	1
43	DNA‐dependent protein kinase catalytic subunit	1
43	AP20 region protein 1	1
42	Protein‐glutamine gamma‐glutamyltransferase E	1
42	Endoplasmin	1
42	Protein FAM149B1	1
39	Desmin	1
39	Lipopolysaccharide‐responsive and beige‐like anchor protein	1
38	Plasminogen activator inhibitor 1	1
37	Lipocalin‐1	1
37	S100‐A8	1
36	Metalloproteinase inhibitor 1	1
36	Serotransferrin	1
35	Junction plakoglobin	1
34	Dimethylaniline monooxygenase [N‐oxide‐forming] 2	1
34	Protein‐glutamine gamma‐glutamyltransferase K	1
33	Solute carrier family 12 member 5	1
33	Ribosomal RNA‐processing protein 8	1
31	Tubulin beta‐3 chain	2
30	KIAA1797	1
30	Ig kappa chain V‐I region Gal	1
30	Ankyrin repeat and SAM domain‐containing protein 4B	1
30	Myosin‐9	1

Proteins were extracted from tissue culture plastic that had been incubated with SAEC‐conditioned medium and induced migration of MCF‐7 cells. Mascot scores and number of different peptides are listed for each protein from the first extraction. All noncontaminating proteins with Mascot scores 30 or greater, primarily cytoplasmic proteins presumably from dead or lysed cells, are listed. Extracellular matrix proteins are in bold.

**Table 2:** Summary of proteins from trypsin (second) extraction detected by mass spectrometry

Mascot score	Protein family	Peptide matches
1091	**Fibronectin**	65
888	**Thrombospondin‐1**	49
303	**Laminin ** ***γ*** **2 chain**	14
292	**Laminin ** ***α*** **3 chain**	18
252	**Laminin ** ***β*** **3 chain**	13
229	Peroxidasin homolog	12
147	**Transforming growth factor‐** ***β*** **‐induced protein ig‐h3**	9
144	Agrin	11
133	Glia‐derived nexin	7
128	**Laminin ** ***γ*** **1 chain**	6
117	Growth/differentiation factor 15	3
86	**Laminin ** ***α*** **5 chain**	5
84	Cadherin‐1	3
80	Actin	5
80	Periostin	1
78	*α*‐enolase	1
73	Tubulin *α*‐1A chain	2
73	Serotransferrin	4
71	Protein CYR61	1
71	Phospholipid transfer protein	4
70	Heat shock protein 70 kDa	9
67	Elongation factor 1*α*1	2
64	Apolipoprotein E	2
57	Coiled‐coil domain‐containing protein 80	2
56	Heat shock protein HSP 90 *α*	3
54	Fructose bisphosphate aldolase A	1
53	Fibroblast growth factor‐binding protein 1	1
52	Collagen *α*‐1 (VII) chain	1
52	Cofillin	1
51	Calsyntenin‐1	1
51	Complement C1q subcomponent subunit A	1
50	Peroxiredoxin‐5, mitochondrial	1
49	Basement membrane‐specific heparan sulfate proteoglycan core protein	1
46	Renin receptor	2
41	A disintegrin and metalloproteinase with thrombospondin motifs 1	2
40	Probable phospholipid‐transporting ATPase IA	1
39	60S acidic ribosomal protein P2	1
39	Rab GDP dissociation inhibitor β	1
39	Matrilin‐3	1
39	Protein FAM40A	1
39	Secretogranin‐2	1
38	Microtubule‐associated protein 1B	1
38	Fructose‐bisphosphate aldolase C	1
38	Urokinase‐type plasminogen activator	1
35	Phosphatidylinositol 3,4,5‐trisphosphate‐dependent Rac exchanger 1 protein	1
34	Insulin‐like growth factor‐binding protein 7	1
34	Phosphatidylethanolamine‐binding protein 1	1
33	Peptidyl‐prolyl cis‐trans isomerase A	2
32	Flavin reductase (NADPH)	1
32	Triosephosphate isomerase	1
32	Craniofacial development protein 1	2
31	Dickkopf‐related protein 3	1
31	**Laminin ** ***β*** **1 chain**	2
30	Vimentin	1
30	Spexin	1

Extracellular matrix proteins are in bold.

**Table 3:** Summary of extracellular matrix proteins detected by mass spectrometry

Mascot score	Mascot score	Protein family	Peptide matches	Peptide matches
First Extraction	Second Extraction		First Extraction	Second Extraction
ND	1091	Fibronectin	N/A	65
308	888	Thrombospondin‐1	11	49
ND	303	Laminin *γ*2 chain	N/A	14
132	292	Laminin *α*3 chain	4	18
97	252	Laminin *β*3 chain	2	13
247	N/D	Tenascin	10	N/A
194	147	TGF‐*β*‐induced protein ig‐h3	8	9
ND	128	Laminin *γ*1 chain	N/A	6
ND	86	Laminin *α*5 chain	N/A	5
ND	31	Laminin *β*1 chain	N/A	2

Proteins were extracted from tissue culture plastic that had been incubated with SAEC‐conditioned medium and induced migration of MCF‐7 cells. Mascot scores and number of different peptides are listed for each protein from both SDS‐deoxycholate (first) and trypsin (second) extractions. Fibronectin, thrombospondin 1, and all three of the laminin 332 chains (*α*3, *β*3, *γ*2) showed the highest Mascot scores and greater numbers of peptide matches. All noncontaminating proteins with Mascot scores 30 or greater are listed in tables S1 and S2. SAEC, small airway epithelial cells; ND, not detected; TGF, transforming growth factor.

### Motility assay of candidates identified by mass spectrometry

**Figure 4:**
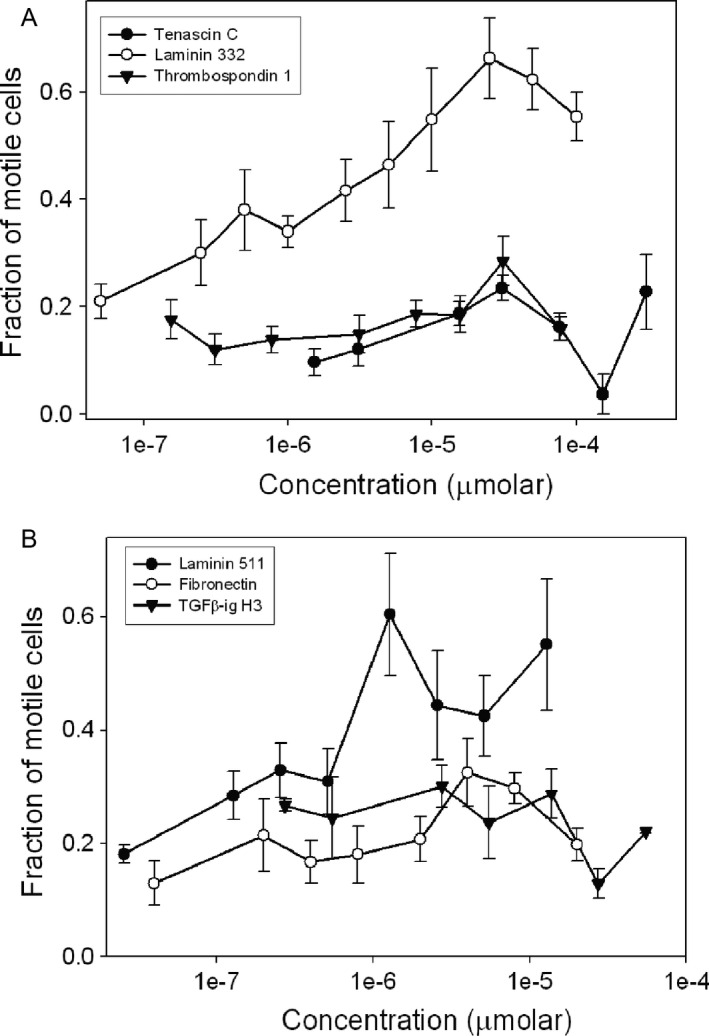
Motility dose–response studies of MCF‐7 cells to extracellular matrix proteins in small airway epithelial cells (SAEC)‐conditioned medium: laminin 332, tenascin C, thrombospondin 1 (A), laminin 511, fibronectin, and TGF *β*‐ig‐h3 (B). Error bars show standard error of the mean.

Motility assays with MCF‐7 cells were performed with purified LM332 and fibronectin, and with recombinant LM511, *β*ig‐h3, tenascin C, and TSP1 at the ranges of concentrations specified in the Materials and Methods section and the results are shown in Figure 4. No migration was observed in response to tenascin C, *β*ig‐h3, fibronectin, or TSP1, but LM332 and LM511 evoked dose‐dependent motility at concentrations of approximately 10^−6^ *μ*mol/L and greater (Fig. 4). Of the two laminins, LM332 showed higher Mascot scores, suggesting that LM332 may be the more highly expressed of the two laminin complexes in the lung CM, and was potentially responsible for most of the MCF‐7 cell motility. Thus, further studies were undertaken to better define the contribution of LM332 to the induction of carcinoma cell motility by SAEC.

### LM332 in SAEC CM and pulmonary epithelium

**Figure 5:**
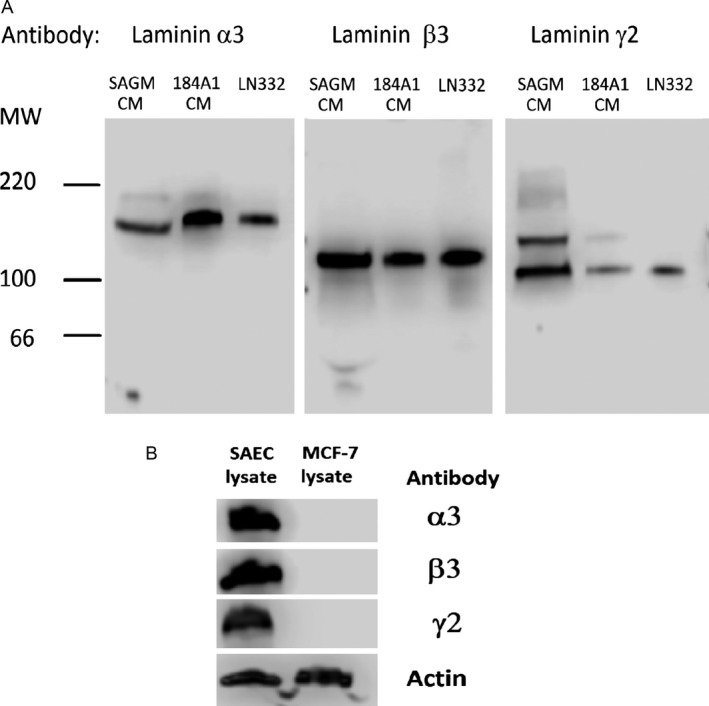
Immunoblot of small airway epithelial cells (SAEC)‐conditioned media probed with LM332 chain antibodies (A). Immunoblot of SAEC and MCF‐7 lysates (B). For each experiment, all lanes were run simultaneously on the same gel.

In order to confirm that LM332 was present among the secretions of SAEC, immunoblotting with antibodies specific for the laminin *α*3, *β*3, and *γ*2 chains was performed. Specific antiserum for human laminin *α*3 chain, or monoclonal antibodies against human *β*3 or *γ*2 chains were incubated with the blots. Conditioned medium for 184A1 mammary epithelial cells and purified LM332 served as positive controls for each reaction. All three chains could be detected at the appropriate molecular weights (Fig. 5A), consistent the presence of LM332 in the CM. The predominant band of the *α*3 chain was approximately 165 kDa, with a minor component at 180 kDa. The *β*3 chain was 140 kDa and the *γ*2 chain was approximately 155 and 105 kDa, The molecular weight bands of the three chains is consistent with previous observations 36. Similarly, lysates of SAEC cells probed with the same antibodies (Fig. 5B) also showed the presence of all three chains in the cytoplasm. An equal amount of MCF‐7 lysate did not show any of the three chains of LM332.

**Figure 6:**
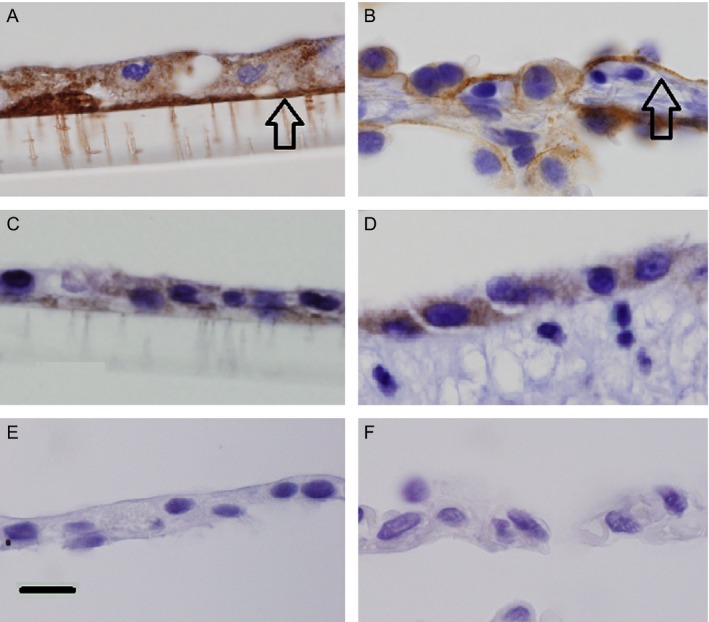
Immunohistochemistry of cultured SAEC on polyester membranes (A,C,E) and human lung (B,D,F) for laminin *β*3 (A, B), and *γ*2 (C, D) chains, and mouse antibody negative controls (E.F). Both chains are expressed in the cytoplasm and basement membranes (arrows) of cultured cells and in tissue. Original magnification 1000×, scale bar = 50 *μ*m. SAEC, small airway epithelial cells; SAGM‐conditioned medium (CM)—conditioned small airway growth medium of SAEC, 184A1 CM conditioned medium of 184A1 cells, LM332‐ laminin 332

In order to verify that LM332 was present in the lung tissue and in the cultured pneumocytes, immunohistochemistry using two different antibodies for LM332 was performed on lung tissue that was used to derive the pneumocytes, and on pneumocytes grown on membranes. The pneumocytes both in the lung tissue and on membranes expressed cytoplasmic LM332 in a similar pattern and formed basement membranes rich in LM332 (Fig. 6).

### Inhibition of motility

**Figure 7:**
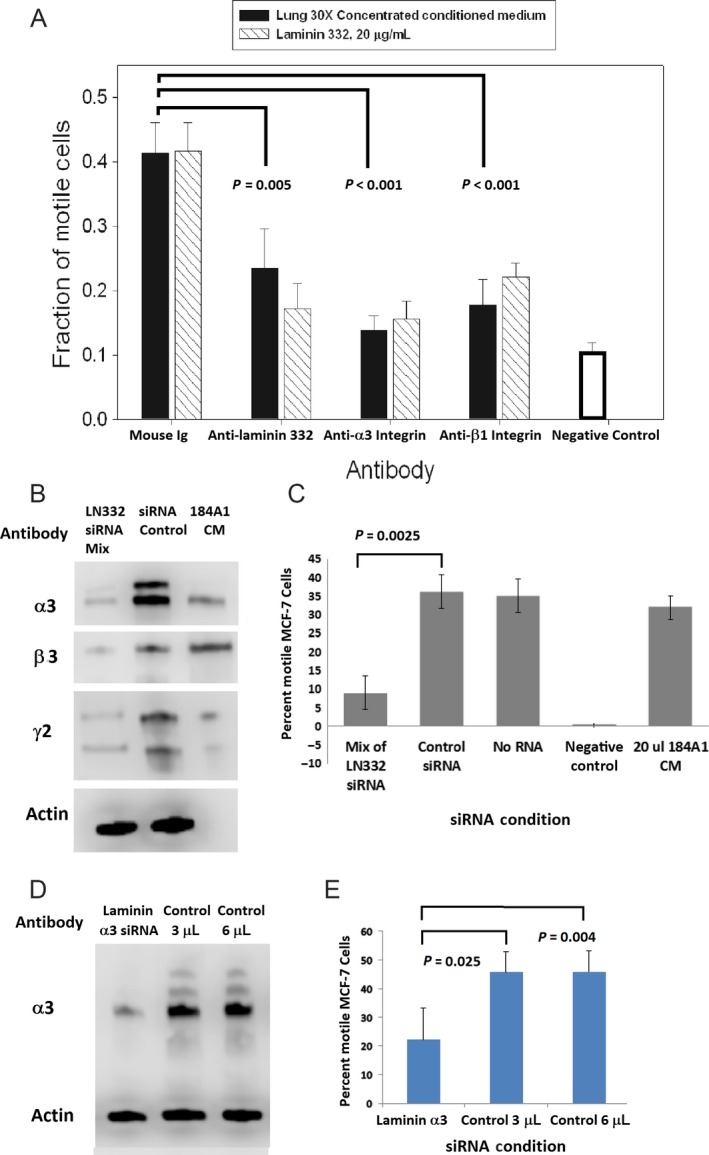
Antibody inhibition of small airway epithelial cells (SAEC)‐induced motility. Antibodies to LM332 and both integrin components significantly inhibited conditioned medium (CM)‐induced motility to the same extent to which they inhibited LM332‐induced motility (A). *P*‐values were determined using one‐way ANOVA with Holm–Sidak correction. Knockdown of LM332. (B). Column 1, (LM332 siRNA mix) refers to lysates of cells transfected with an equal amount of each of the siRNA directed against the three chains of laminin 332 in one reaction. Column 2 (siRNA Control) has a nonspecific sequence, in a concentration equal to the total siRNA mix, Column 3 (184A1 CM) provides a positive LM332 control. Motility of MCF‐7 cells treated with conditioned media from SAEC transfected with laminin chain siRNA or control siRNA (C), showing significantly reduced motility from CM of siRNA‐treated SAEC cells. siRNA against the *α*3 chain only on a immunoblot reacted with both anti‐*α*3 and actin antibodies (D). The *α*3 siRNA resulted in CM with a reduced ability to induce MCF‐7 migration (E), while the control sequence in the same or double the siRNA concentration did not knockdown *α*3 chain expression (D) and their CM did not affect motility (E). Error bars show standard errors of the mean. For each experiment, all lanes were run simultaneously on the same gel.

As a further confirmation that LM332 was the pulmonary motility factor, LM332 and components of lung CM, each bound to separate tissue culture plate wells, were treated with blocking antibodies to LM332 and the components of *α*3*β*1 integrin, the primary receptor for LM332 motility on MCF‐7 cells 16, 37. (Fig. 7A). In the presence of mouse antibody control (left bars), SAEC‐conditioned medium and LM332 induced MCF‐7 motility to similar degrees. Using the same amounts of LM332 and SAEC as were used for controls, inhibiting antibodies to LM332, *α*3 integrin, and *β*3 integrin equivalently reduced both LM332 and SAEC‐induced motility to levels similar to baseline migration in the absence of either LM332 or SAEC CM (right bar). The difference in motility between MCF‐7 cells treated with SAEC CM and antibodies to LM332, *α*3 integrin, and *β*3 integrin antibodies compared to control is significant (*P* = 0.005, <0.001, and <0. 001, respectively).

Finally, LM332 expression in SAEC was knocked down with siRNA, and the CM of the SAEC was used to test for motility in MCF‐7. In order to knockdown the complete LM332 trimer in SAEC, siRNA against the *α*3, *β*3, and *γ*2 chains were mixed together to transfect SAEC. After a 48 h incubation with the RNA and transfection reagent, the CM of the transfected cells was used for motility assays, and the cell lysates were tested for protein expression of each of the chains by immunoblotting. A majority of each chain's expression was knocked down compared to control siRNA (Fig. 7B). There was less motility induction in MCF‐7 cells from the CM of the cells transfected with the siRNA of the three LM332 chains (Fig. 7C). The experiment was repeated using siRNA against *LAMA3* only, resulting in almost complete knockdown of the *α*3 chain of the protein as determined by immunoblot (Fig 7D). In the lysates used for the immunoblot, the completely processed form of the protein predominates. Significantly decreased motility was induced in MCF‐7 cells using the CM of the SAEC treated with siRNA against the *α*3 chain compared to controls with the same or double the siRNA concentration (Fig 7E). This finding is consistent with observations that the globular domains of the *α*3 laminin chain are likely primary contributors to the migration‐ inducing effect of LM332 38.

## Discussion

In this study, we investigated whether lung epithelium had the ability to induce tumor cell migration. In order to test this hypothesis, MCF‐7 breast carcinoma cells were exposed to SAEC, which resulted in tumor cell migration. MCF‐7 was used for this study since it does not produce LM332 itself (Fig. 5B) and does not migrate in normal culture conditions, allowing only the effect of exogenous motility factors to be measured. Our previous work has validated that scattering is a motile response by time‐lapse video microscopy and comparison with Boyden chamber assays 26, 27.

CM from the cultured SAEC revealed that at least one of the factors responsible for this behavior was secreted by the SAEC and could induce motility after binding to tissue culture plastic. Among the most abundant proteins on the plastic identified by mass spectrometry, and implicated in tumor cell motility in previous studies 16, 17, LM332 had the greatest migration‐inducing effect on MCF‐7 cells. All three of the LM332 chains were present in SAEC CM, and IHC for LM332‐specific chains allowed its identification in the lung and in SAEC. Antibodies to either LM332 or its integrin receptor reduced this motility, as did knockdown of the three LM332 chains with siRNA. Taken together, these findings implicate LM332 as a material residing in the lung tissue that has the potential to induce motility in breast carcinoma cells. The only situation in which pulmonary LM332 could influence breast carcinoma migration, however, is when breast cancer cell metastasize to the lung.

Since tumor cell migration is essential for metastasis to occur 3, 4, this suggests that LM332 has the potential to promote metastasis to the lung. There is evidence that LM332 may play a role in metastasis 30, 39, 40, 41, 42. For example, one study showed that knockdown of laminin 5 in A549 lung carcinoma cell spheroids by shRNA decreased pulmonary metastasis, and positive staining of LM332 in human lung cancer was associated with a worse prognosis 39. In other investigations, anti‐LM332 antibodies suppress metastases of LM332‐expressing squamous carcinoma, 40 and knockdown of genes expressing either the laminin *β*3 41 or *γ*2 42 chains of LM332 (*LAMB3, LAMC2,* respectively) in lung carcinoma cells decreases their metastatic potential. LAMC2 is overexpressed in A549 cells that have been selected for high metastatic potential compared to nonselected cells 42. Some breast carcinomas, such as metaplastic and estrogen receptor (ER)‐negative cancers express LM332 30, 43, however, most breast carcinomas do not 44. Thus, LM332 in the microenvironment is more likely to play a role in breast carcinoma progression than LM332 from the breast carcinoma cells themselves. This notion is supported by observations that microenvironmental LM332 in breast tissue can potentially stimulate tumor invasion 16, 30, 45. The findings presented here indicate that LM332 is not only present in the lung tissue, but that the LM332 in the lung has the potential to induce migration of breast cancer cells, providing a means for them to enter the pulmonary parenchyma and establish a new colony of tumor cells.

Other findings in the literature are consistent with the possibility that LM332 in the lung tissue could contribute to tumor metastasis. LM332 in mouse lung has been identified 35, consistent with our findings in human tissue, and Wang et al. reported that HT1080 fibrosarcoma cells adhere to LM332 on endothelium in pulmonary capillaries, providing a role for arrest of tumor cells prior to the establishment of a metastatic colony 46. In contrast to those findings, however, we did not identify LM332 in pulmonary endothelium by IHC, and we examined the migratory rather than the adhesive role of LM332 in our study. These investigators also provided evidence that tumor cell arrest was mediated by the expression of *α*3*β*1 integrin on the tumor cells. Although there is conflicting evidence on whether *α*3*β*1 integrin mediates metastasis in all cases, *α*3*β*1 integrin 47 and LM332 48 have been proposed as antimetastatic targets, and additional experimental evidence of an antitumor response by an anti‐LM332 antibody has been reported 49. Unlike earlier studies that targeted LM332 expression on subcutaneous 40, 49 or lung 40, 42 tumors, our study examined the LM332 from normal tissue. This raises the possibility that blocking LM332 could inhibit the pulmonary metastasis of a tumor that does not express LM332.

In summary, our findings are consistent with the known role of LM332 as a motility factor and provide evidence that LM332 can act within the microenvironment rather than in tumor cells alone. Furthermore, the origin of the microenvironmental signal can originate from epithelial cells.

The intent of this study was to confirm that pulmonary LM332 has properties sufficient to enhance tumor cell migration and as a rationale to proceed to animal studies. Demonstration that pulmonary LM332 can actually lead to metastasis will require an in vivo model. Based on our findings, as well as other studies of metastasis in the literature 2, 46, animal studies can then be used to test the following hypothesis. First, metastatic tumor cells arrest in pulmonary microvasculature, either by size exclusion or by endothelial adhesion. After extravasation, which may be facilitated by endothelial and other cell types 15, tumor cells enter the extravascular space. It is here where they come in contact with pulmonary epithelial cell basement membrane in the extracellular space and can spread along the pulmonary basement membrane in response to LM332. Furthermore, LM332 may support the growth of some epithelial cancers 50, or promote tumor cell adhesion to the ECM, in turn increasing their chance of survival.

Most likely, a process as complex as metastasis requires numerous contributing events, both originating in the cancer cells themselves, and also from the tumor microenvironment. Additional motility factors from the microenvironment may be identified with examination of different model systems. Further study will be needed to determine whether LM332 from pulmonary epithelial cells is involved in the progression of metastasis in an in vivo model, and if this is a component of the metastatic cascade that is vulnerable to attack as a target of novel therapeutic agents.

## Conflict of interest

PMC is a supplier of purified laminin 332 to Kerafast, Inc. The other authors have no disclosures.
